# S-allyl-cysteine triggers cytotoxic events in rat glioblastoma RG2 and C6 cells and improves the effect of temozolomide through the regulation of oxidative responses

**DOI:** 10.1007/s12672-024-01145-3

**Published:** 2024-07-08

**Authors:** Carolina Y. Reyes-Soto, Ricardo J. Ramírez-Carreto, Luz Belinda Ortíz-Alegría, Alejandro Silva-Palacios, Cecilia Zazueta, Sonia Galván-Arzate, Çimen Karasu, Isaac Túnez, Alexey A. Tinkov, Michael Aschner, Tessy López-Goerne, Anahí Chavarría, Abel Santamaría

**Affiliations:** 1 Posgrado en Ciencias Biológicas, Universidad Nacional Autónoma de México, 04510 Mexico City, Mexico; 2 Unidad de Investigación en Medicina Experimental, Facultad de Medicina, Universidad Nacional Autónoma de México, 06726 Mexico City, Mexico; 3 Facultad de Química, Universidad Nacional Autónoma de México, 04510 Mexico, Mexico; 4 Programa de Doctorado en Ciencias Biomédicas, Universidad Nacional Autónoma de México, 04510 Mexico City, Mexico; 5 Laboratorio de Inmunología Experimental, Subdirección de Medicina Experimental, Instituto Nacional de Pediatría, Secretaría de Salud, 04530 Mexico City, Mexico; 6 Departamento de Biomedicina Cardiovascular, Instituto Nacional de Cardiología Ignacio Chávez, SSA, 14080 Mexico City, Mexico; 7 Departamento de Neuroquímica, Instituto Nacional de Neurología y Neurocirugía Manuel Velasco Suárez, S.S, 14269 Mexico City, Mexico; 8 Department of Medical Pharmacology, Cellular Stress Response and Signal Transduction Research Laboratory, Faculty of Medicine, Gazi University, 06500 Ankara, Turkey; 9 Departamento de Bioquímica y Biología Molecular, Facultad de Medicina y Enfermería, Instituto de Investigaciones Biomédicas Maimónides de Córdoba (IMIBIC)Universidad de CórdobaRed Española de Excelencia en Estimulación Cerebral (REDESTIM), 14071 Córdoba, Spain; 10 Laboratory of Molecular Dietetics, IM Sechenov First Moscow State Medical University (Sechenov University), Moscow, 119435 Russia; 11 Departament of Elementology, and Department of Human Ecology and Bioelementology, Peoples’ Friendship University of Russia (RUDN University), Moscow, 117198 Russia; 12 Laboratory of Molecular Ecobiomonitoring and Quality Control, Yaroslavl State University, Yaroslavl, 150003 Russia; 13 Department of Molecular Pharmacology, Albert Einstein College of Medicine, Bronx, NY 10461 USA; 14 Laboratorio de Nanotecnología y Nanomedicina, Departamento de Atención a la Salud, Universidad Autónoma Metropolitana-Xochimilco, 04960 Mexico City, Mexico; 15 Facultad de Ciencias, Universidad Nacional Autónoma de México, 04510 Mexico City, Mexico

**Keywords:** Glioblastoma, Redox modulation, Nrf2-ARE, Inflammatory cytokines, Cytotoxicity, RG2 rat cells, C6 rat cells, S-allyl-cysteine

## Abstract

Glioblastoma (GBM) is an aggressive form of cancer affecting the Central Nervous System (CNS) of thousands of people every year. Redox alterations have been shown to play a key role in the development and progression of these tumors as Reactive Oxygen Species (ROS) formation is involved in the modulation of several signaling pathways, transcription factors, and cytokine formation. The second-generation oral alkylating agent temozolomide (TMZ) is the first-line chemotherapeutic drug used to treat of GBM, though patients often develop primary and secondary resistance, reducing its efficacy. Antioxidants represent promising and potential coadjutant agents as they can reduce excessive ROS formation derived from chemo- and radiotherapy, while decreasing pharmacological resistance. S-allyl-cysteine (SAC) has been shown to inhibit the proliferation of several types of cancer cells, though its precise antiproliferative mechanisms remain poorly investigated. To date, SAC effects have been poorly explored in GBM cells. Here, we investigated the effects of SAC in vitro, either alone or in combination with TMZ, on several toxic and modulatory endpoints—including oxidative stress markers and transcriptional regulation—in two glioblastoma cell lines from rats, RG2 and C6, to elucidate some of the biochemical and cellular mechanisms underlying its antiproliferative properties. SAC (1–750 µM) decreased cell viability in both cell lines in a concentration-dependent manner, although C6 cells were more resistant to SAC at several of the tested concentrations. TMZ also produced a concentration-dependent effect, decreasing cell viability of both cell lines. In combination, SAC (1 µM or 100 µM) and TMZ (500 µM) enhanced the effects of each other. SAC also augmented the lipoperoxidative effect of TMZ and reduced cell antioxidant resistance in both cell lines by decreasing the TMZ-induced increase in the GSH/GSSG ratio. In RG2 and C6 cells, SAC per se had no effect on Nrf2/ARE binding activity, while in RG2 cells TMZ and the combination of SAC + TMZ decreased this activity. Our results demonstrate that SAC, alone or in combination with TMZ, exerts antitumor effects mediated by regulatory mechanisms of redox activity responses. SAC is also a safe drug for testing in other models as it produces non-toxic effects in primary astrocytes. Combined, these effects suggest that SAC affords antioxidant properties and potential antitumor efficacy against GBM.

**Supplementary Information:**

The online version contains supplementary material available at 10.1007/s12672-024-01145-3.

## Introduction

Glioblastoma (GBM) is an aggressive form of brain cancer originating from astrocytic cells with worldwide annual incidence of 0.59–3.69 per 100,000 persons. Among the more than 100 different types of neoplasia affecting human, GBM is one of the most resistant to treatment, representing the most frequent and aggressive tumor of the Central Nervous System (CNS) [1, 2]. GBM is characterized by anaplasia, nuclear atypia, cellular pleomorphism, mitotic activity, and, most remarkably, alternated phases of rapid proliferation and aggressive invasion of the surrounding brain tissue, which inevitably leads to critical recurrence after surgical resection of the main tumor mass [3, 4]. The first therapeutic approach for GBM is surgery, followed by radio- and chemotherapy. Despite important advances in these two approaches, glioma cells may still invade neighboring tissues beyond their detection, leading to tumor recurrence. In general terms, prognosis is not favorable as approximately half of the patients die within the first year after diagnosed, the vast majority of which die within the first two years, and less than 5% survive for 5 years [5]. GBM tumors are classified as primary or secondary; approximately 90% of cases are primary and occur de novo in elderly patients, whereas secondary cases progress from lower-grade astrocytoma, and are more prevalent in younger patients. This type of cancer affects intellectual, cognitive and physical skills, seriously compromising the quality of life of patients. Cancer progresses as the patient gradually loses neurological functions and autonomy. Unfortunately, death occurs after an intense and short-term loss of neurological function [6].

In the tumor microenvironment (TME), glioma cells face several challenges, including hypoxia, acidity, and limited nutrients’ availability. To maintain rapid growth, tumor cells must adapt to these severe biochemical changes and modify their metabolic activity by augmenting glycolysis (Warburg effect), thus producing increased amounts of lactic acid [7]. This condition provides cancer cells with the advantage of being independent of oxygen as a primary source of energy, especially in adverse tumor microenvironment, in turn leading to prolonged survival and drug resistance [8]. Thus, the development of adaptive strategies to regulate metabolic alterations, angiogenesis, and migration is essential for cancer cells to survive metabolic stress and ensure an optimum nutrient supply as tumor mass accumulates.

Reactive Oxygen Species (ROS) are reactive byproducts derived from the partial reduction of oxygen that are produced mainly by mitochondria and endothelial enzymes. Under normal conditions, appropriate functioning of the redox systems prevents oxidative damage to biomolecules in cells and tissues; however, when ROS levels surpass the antioxidant defense systems, oxidative stress (OS) occurs. Oxidative damage in cells is tightly related with tumorigenesis. During the first steps of cancer development, an intrinsic increase in ROS formation is related to a wide spectrum of pathophysiological activities, such as oncogene activation, enhanced metabolism, and mitochondrial alterations [9]. Moreover, through the generation of ROS, particularly of hydrogen peroxide, tumor cells can damage other cells and tissues, thus facilitating tumor growth and invasiveness [10]. During the progression stage, the increase in ROS formation can modulate several signaling pathways and activate transcription factors such as the nuclear factor erythroid 2-related factor 2 (Nrf2) and the nuclear factor κB (NFκB) to adapt and promote “a redox reestablishment” [11] aimed to control stability, cell survival, and metabolic adaptation, promoting drug resistance [12–14]. Inflammatory events lead to tumorigenesis as a result of excessive ROS and Reactive Nitrogen Species (RNS) formation secreted by macrophages and other cells in the immune system, thus highlighting the relationship between OS and inflammation.

The failure of therapeutic approaches for GBM which have shown efficacy for other types of cancer highlights the challenges in treating brain tumors. The second-generation oral alkylating agent temozolomide (TMZ) is the first-line chemotherapeutic drug used against GBM due to its ability to cross the blood–brain barrier. TMZ affects single DNA strands at specific sites and preferentially methylates DNA at the O6 position of guanine [15]. Alkylation of the guanine O6 site leads to the formation of O6-methylguanine and the consequent insertion of thymine residues at the cytosine site. These irreparable mutations induce single- and double-stranded DNA breaks, leading to cell cycle arrest at the G2/M phase and apoptosis. Despite the use of TMZ for the treatment of GMB has shown some advantages, such as moderate side effects and increased life expectancy in patients, GBM frequently develops primary and secondary resistance to this drug due to the overexpression of O6-methylguanine-DNA-methyltransferase (MGMT) protein, which can repair the DNA damage induced by TMZ, thus preventing apoptosis of cancer cells and favoring tumor recurrence [15]. Conventional therapies against cancer often face obstacles due to drug resistance [16] and severe side-effects at the systemic level related to excessive ROS formation caused by exposure to chemo- and radiotherapy [17]; consequently, biomedical sciences are in search for alternatives that overcome pharmacological resistance and reduce side-effects using complementary approaches (Complementary and Alternative Medicine, or CAM) [18]. While important advances in the treatment of several types of cancer have been achieved, the improvement of GBM patients shows only moderate advances [1]. Therefore, the exploration of novel and promising therapies at the experimental level endorses major relevance.

Natural compounds derived from food have been investigated as novel sources of potential antitumor compounds [19, 20] as several of them act in a similar manner to conventional chemotherapeutic compounds by arresting the cell cycle and inducing apoptosis. Several studies demonstrated that organic sulfur compounds naturally located in garlic, such as S-allyl-cysteine (SAC), are potentially responsible for the decreased risk of developing cancer due to their chemopreventive effects achieved at low doses [21]. However, there are reports suggesting that typical antioxidant compounds may also induce pro-oxidant activity, accounting for their chemotherapeutic potential at high doses [22]. In this regard, SAC and garlic-derived compounds, at high doses, are capable of inhibiting tumor cell proliferation while inducing apoptosis in cell lines of human prostate, colon, gastric, ovarian, and breast cancers, as well as in neuroblastoma [23–28]. Both antioxidant and chemopreventive properties have been reported for SAC, including its capacity to neutralize ROS/RNS, chelate Fe^2+^ and Cu^2+^, and reduce the Fenton reaction.

Further, SAC stimulates the synthesis of enzymatic and non-enzymatic antioxidants by activation of Nrf2, the master regulator of cell redox homeostasis [29]. Nrf2 coordinates the up-regulation of antioxidant gene expression when complexed with the Antioxidant Response element (ARE) at the DNA, thus orchestrating a defensive response against xenobiotics and antioxidants referred to as Phase II response, recruiting enzymes such as heme oxygenase-1 (HO-1), glutathione S-transferase (GST), and glutathione peroxidase (GPX), among several others [30]. During progression and treatment, cancer cells may develop adaptive responses to OS induced by chemotherapeutic agents through the constitutive activation of Nrf2 [22].

Despite antiproliferative and proapoptotic properties of SAC have already been described in tumor cells, the precise mechanisms and recruited signaling pathways leading to these effects remain poorly explored. These mechanisms may exert important antitumor effects. Therefore, in this study we investigated and compared the cytotoxic effects of SAC in vitro in two rat GBM cell lines to elucidate some of the molecular and cellular mechanisms induced by this natural compound, as well as its role in the modulation of oxidative processes mediated by the transcription factor Nrf2. We also investigated whether SAC exerted an additive effect with TMZ to validate its potential as coadjutant therapy.

## Materials and methods

### Glioblastoma cell lines culture

The rat glioblastoma cell lines RG2 and C6 were used; both were kindly provided by Drs. Alette Ortega and Elizabeth Ortiz from the Instituto Nacional de Cancerología (INCAN), Mexico. Cells were cultured in Eagle’s minimal essential medium modified by Dulbecco’s medium (high-glucose DMEM; Invitrogen Co., Grand Island, NY, U.S.A.) and supplemented with 10% fetal bovine serum (FBS; Gibco, Grand Island, NY, U.S.A.) and 1 mL of 1% penicillin–streptomycin (Invitrogen Co., Grand Island, NY, U.S.A.) in a humidified atmosphere of 95% air and 5% CO_2_ at 37 °C. All reagents were of analytical grade and were obtained from well-known reagent companies. In parallel, primary astrocyte cells were cultured and exposed to similar experimental conditions for comparison with glioma cells (Supplementary Material).

### Assessment of cell viability

This assay is based on the metabolic reduction of 3-(4,5-dimethylthiazol-2-yl)-2,5-diphenyltetrazolium bromide (MTT) to formazan by the mitochondrial enzyme succinate dehydrogenase in the presence of NADPH [31]. The assay is optimum for the assessment of cell viability in cultured cells, as the optical density detecting color of the reaction is directly proportional to the number of viable mitochondria.

SAC and TMZ were dissolved in PBS. Cells were seeded in 96 well-plates in high-glucose DMEM containing 10% FBS at an initial density of 1 × 10^4^ cells/well and incubated for 24 h at 37 ºC in a 5% CO_2_ atmosphere. Next, cells were treated with increased concentrations of SAC (1–750 µM) and/or TMZ (100–1000 µM) and incubated for 48 h at 37 ºC. After 48 h, 15 µl of the MTT reagent (0.1 mg/mL final concentration) was added and samples were incubated for 3 h at 37 ºC. After incubation, the medium was removed and formazan crystals were solubilized in 100 µL isopropanol. The amount of formazan was estimated at 570 nm wavelength using a microplate reader (Cytation, BioTek, Winooski, Vermont, U.S.A.). Results are expressed as the percentage of MTT reduction *vs.* the control values (n = 4 experiments per group). This assay was also carried out in primary astrocytes to determine cell viability in non-tumor cells (*Supplementary Material*).

### Estimation of lipid peroxidation by the Thiobarbituric Acid-Reactive Substances (TBARS) assay

Lipid peroxidation (LPO) is a toxic event accompanying cellular lesions and results from the biological effects of ROS/RNS on lipid substrates. Consequently, detection of its products represents a useful endpoint for estimating the degree of oxidative stress. The most simple and common method to estimate LPO is the measurement of malondialdehyde (MDA), a byproduct originated during the last stages of this toxic event that can be detected as a thiobarbituric acid-reactive substance (TBARS) [31, 32]. This method is based on the reaction of MDA with thiobarbituric acid (TBA), which forms a pink chromophore that can be measured by spectrophotometry at 532 nm. MDA concentration was directly proportional to the detected optical density.

Briefly, cells were seeded in 24 well-plates in high-glucose DMEM containing 10% FBS at an initial density of 4 × 10^4^ cells/well and incubated for 48 h at 37 ºC in a 5% CO_2_ atmosphere. After incubation with SAC (1 µM or 100 µM), TMZ (500 µM) or TMZ + SAC at different concentrations, media were aspired, and cells were homogenized in lysis buffer; 100 µl-aliquots of the samples were added to 50 µl of the TBA reagent (0.75 g TBA + 15 g trichloroacetic acid + 2.53 ml of HCl) and incubated in a shaking water bath at 94 ºC for 20 min. Next, samples were centrifuged at 3,000 g for 10 min at 4 ºC. Supernatants were collected, and the optical density was recorded at 532 nm in a Cytation 3 Imaging Reader (BioTek). A standard curve constructed with increased concentrations of 1,1,3,3-tetramethoxypropane (TMPO) was used to calculate TBARS. Results are expressed as the percentage of TBARS formed (originally calculated as nmol/mg protein) *vs.* control values (n = 4 experiments per group).

### Reduced/oxidized glutathione (GSH/GSSG) detection assay

GSH and GSSG were detected using a method previously described [33], with modifications [31]. This method is based on the ability of o-phthalaldehyde (OPA) to react with primary amines in the presence of a thiol group to generate a fluorescent compound (isoindole), which can be detected at excitation and emission wavelengths of 420 nm and 350 nm, respectively. The reaction of GSH with OPA occurred at pH 8.0, whereas GSSG reacted with OPA at pH 12. Since a pH above 8.0 produces the oxidation of GSH, N-ethylmaleimide (NEM) was added to maintain pH conditions. NEM inhibits glutathione reductase enzyme. Following the treatment of cells with SAC (1 µM or 100 µM), TMZ (500 µM), or TMZ + SAC, 250 µl of lysis buffer was added, and samples were stored at −70 °C. Aliquots of 50 µl were separated for the protein quantification using Lowry’s assay. To quantify GSH, two dilutions were used, the first one consisted of 50 µl sample + 450 µl of DMEM medium were carried out; next, 100 µl of the dilutions were added to 1.8 ml phosphate buffer + 100 µl OPA and incubated for 15 min at room temperature. GSSG was quantified using two sequential dilutions; the first one consisted of 50 µl of sample + 450 µl of DMEM medium, and the second dilution consisted of 100 µl of the first dilution + 200 µl of 0.04 M NEM + 4.3 ml of NaOH and incubated for 30 min. Then, 100 µl was added to 1.8 ml of phosphate buffer + 100 µl OPA and incubated for 15 min at room temperature. Total glutathione content in samples was determined by comparing the optical density obtained from the samples with a standard curve generated from known GSH and GSSG concentrations. The glutathione content was estimated for each sample as nmol/mg protein. Final expression of results is presented as the percentage of GSH or GSSG compared to the control, where the control was considered 100% (n = 4 experiments per group).

### Isolation of cytosolic and nuclear fractions

Cells were preserved at −70 ºC until used for the obtention of nuclear fractions using the NE-PER kit (Thermo Scientific, Rockford, IL, U.S.A.), following the instructions of the manufacturer and supplementing the preparations with a protease inhibitor (Mini-cocktail of protease inhibitors, Roche, Germany) and stored again at −70 ºC.

### Nrf2/ARE binding assay

Nrf2 activity was determined using the assay TransAm Nrf2 (Active Motif, Carlbad, CA, U.S.A.), according to a previously described method [34]. Nuclear extracts (10 μg) were incubated with immobilized oligonucleotides containing the ARE consensus sequence at the binding site (5’-GTCACAGTGACTCAGCAGAATCTG-3') in 96 well-plates. The active form of Nrf2 bound to the oligonucleotide was detected using a primary antibody against Nrf2 after treatment with a secondary conjugated HPR antibody. Nrf2 activity is dependent on both the binding of nuclear extracts with the ARE sequence (embedded at the bottom of the well) and the formation of the colored complex (Ac 1º-Ac 2º coupled to HRP and TMB). To analyze binding, optical density was recorded at 450 nm, using a Synergy multimode plate reader (BioTek, Winooski, VT, U.S.A.). Absorbance was graphically expressed as Nrf2/ARE binding activity (O.D. 450 nm; n = 4 experiments per group).

### Statistical analysis

Data are shown as mean values ± SEM of n = 4 independent experiments per group, each in duplicate. Results were analyzed by one-way analysis of variance (ANOVA) followed by Bonferroni’s test for multiple comparisons, using Prism software (version 6.0; GraphPad, San Diego, CA, U.S.A). Differences were considered as statistically significant at *p* ≤ 0.05.

## Results

### SAC administration decreased the cell viability of RG2 and C6 cell lines

**Fig. 1:**
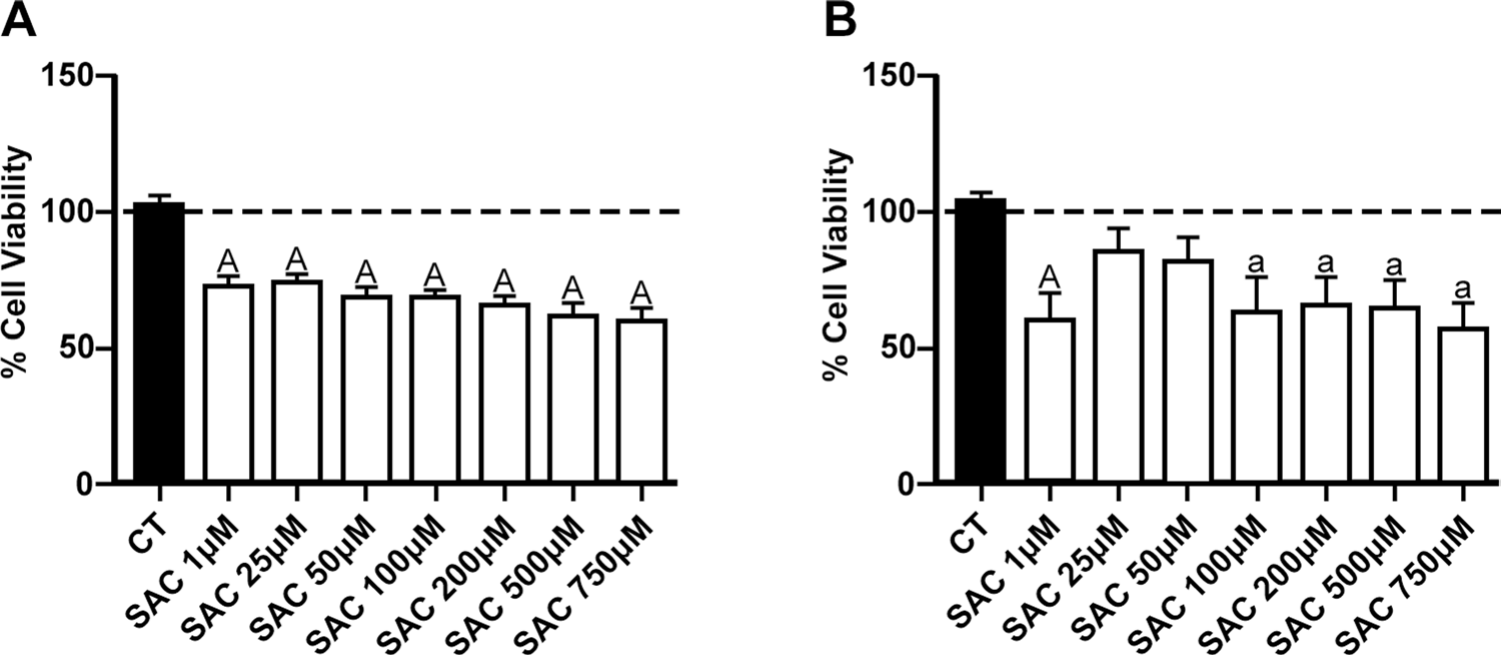
Effect of S-allyl-cysteine (SAC) on cell viability of RG2 and C6 cell lines. Cells were treated with increased concentrations of SAC (1 µM, 25 µM, 50 µM, 100 µM, 250 µM, 500 µM and 750 µM) for 48 h. Charts depict the percentage of cell viability (compared to the control) of RG2 (**A**) and C6 (**B**) cells. Bars represent mean values ± S.E.M. of four experiments per group, each in duplicate. ^a^*p* ≤ 0.05 and ^A^*p* ≤ 0.01, different of control (PBS in culture medium). One-way ANOVA followed by Bonferroni’s test

Cell viability was estimated in tumor cells using the MTT reduction assay. To determine the sensitivity of RG2 and C6 to SAC, the concentration-dependent effect of SAC was explored (1–750 µM). Figure 1 depicts the concentration-dependent effects of SAC on both cell lines. Figure 1A shows that both low and high SAC concentrations significantly decreased RG2 cell viability (*p* ≤ 0.05-*p* ≤ 0.01) compared to the control group (~ 25 to 50% decrease), whereas Fig. 1B depicts a biphasic effect of SAC on C6 cells’ viability observed in a low range of micromolar concentrations (1–50 µM; *p* ≤ 0.01), with significant loss of viability at 100–750 µM (~ 45 to 50% decrease).

### TMZ administration decreased the cell viability of RG2 and C6 cell lines

**Fig. 2:**
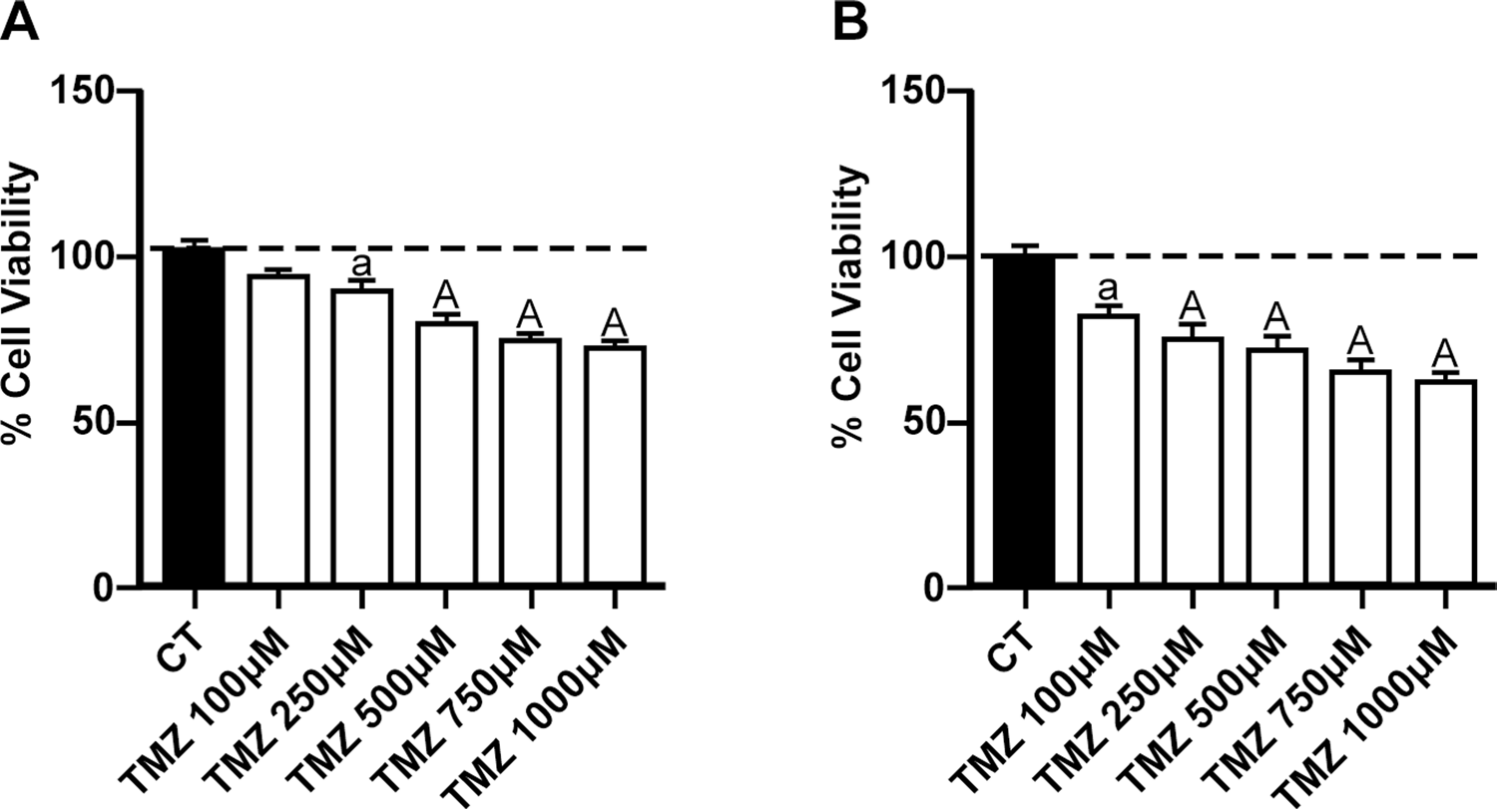
Effect of temozolomide (TMZ) on cell viability in RG2 and C6 cell lines. Cells were treated with increased concentrations of TMZ (100–1000 µM) for 48 h. Charts depict the percentage of cell viability (compared to the control) of RG2 (**A**) and C6 (**B**) cells. Bars represent mean values ± S.E.M. of four experiments per group, each in duplicate. ^a^*p* ≤ 0.05 and ^A^p ≤ 0.01, different of control (PBS in culture medium). One-way ANOVA followed by Bonferroni’s test

To determine the sensitivity of RG2 and C6 cells to TMZ, the concentration–response of this compound was explored at concentrations ranging from 100 to 1000 µM (Fig. 2). Figure 2A shows the concentration-dependent effect of TMZ on RG2 cell viability, reaching a maximum effect between 750 and 1000 µM (~ 25 to 30% decrease; *p* ≤ 0.01, compared to the control group). A similar effect on cell viability was observed in C6 cells (Fig. 2B), with a maximum effect between 750 and 1000 µM (~ 40 to 45% decrease; *p* ≤ 0.01, compared to the control group).

### The combination of SAC + TMZ improves the effects of both compounds on cell viability of RG2 and C6 cell lines

**Fig. 3:**
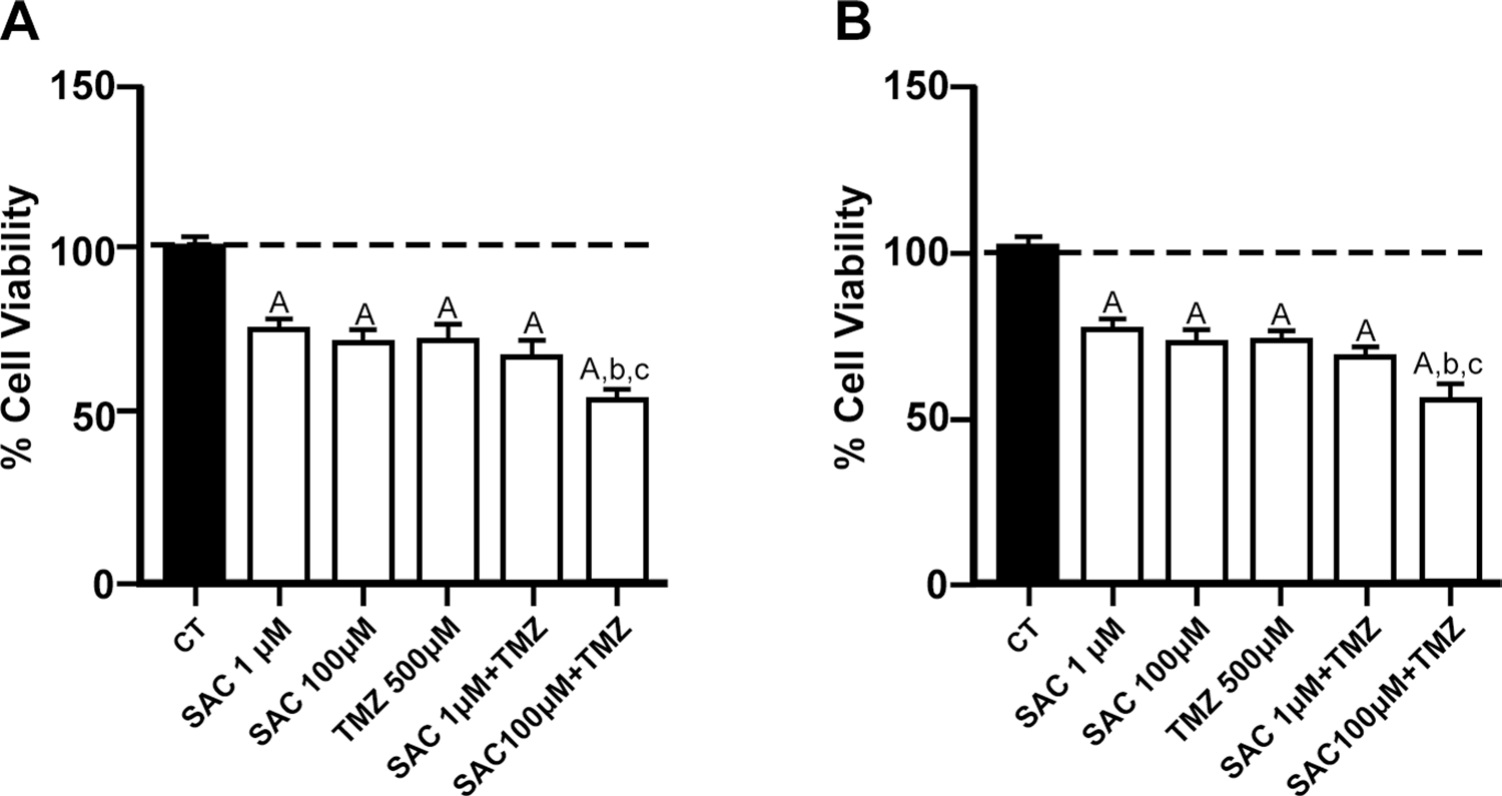
Additive effect of SAC + TMZ on cell viability in RG2 and C6 cell lines. Cells were treated with SAC (1 µM and 100 µM) and/or TMZ (500 µM) for 48 h. Charts depict the percentage of cell viability (compared to the control) of RG2 (**A**) and C6 (**B**) cells. Bars represent mean values ± S.E.M. of four experiments per group, each in duplicate. ^A^*p* ≤ 0.01, different of control (PBS); ^b^*p* ≤ 0.05, different of SAC (1 µM); ^c^*p* ≤ 0.05, different of SAC (100 µM); ^d^*p* ≤ 0.05, different of TMZ. One-way ANOVA followed by Bonferroni’s test

The MTT reduction assay was carried out also in RG2 and C6 cells exposed to SAC + TMZ to investigate the possible additive effect of these two compounds (Fig. 3). Results revealed that these compounds reduced the viability of both cell lines when administered separately (1 or 100 µM SAC and 500 µM TMZ; *p* ≤ 0.01) compared to the control values; when 100 μM SAC was combined with 500 μM TMZ, both RG2 (Fig. 3A) and C6 (Fig. 3B) decreased viability more prominently than both compounds administered separately (*p* ≤ 0.05, compared to SAC and TMZ separately). Both cell lines exhibited the same sensitivity to the experimental conditions tested.

### TMZ and SAC + TMZ increase lipid peroxidation in RG2 and C6 cell lines

**Fig. 4:**
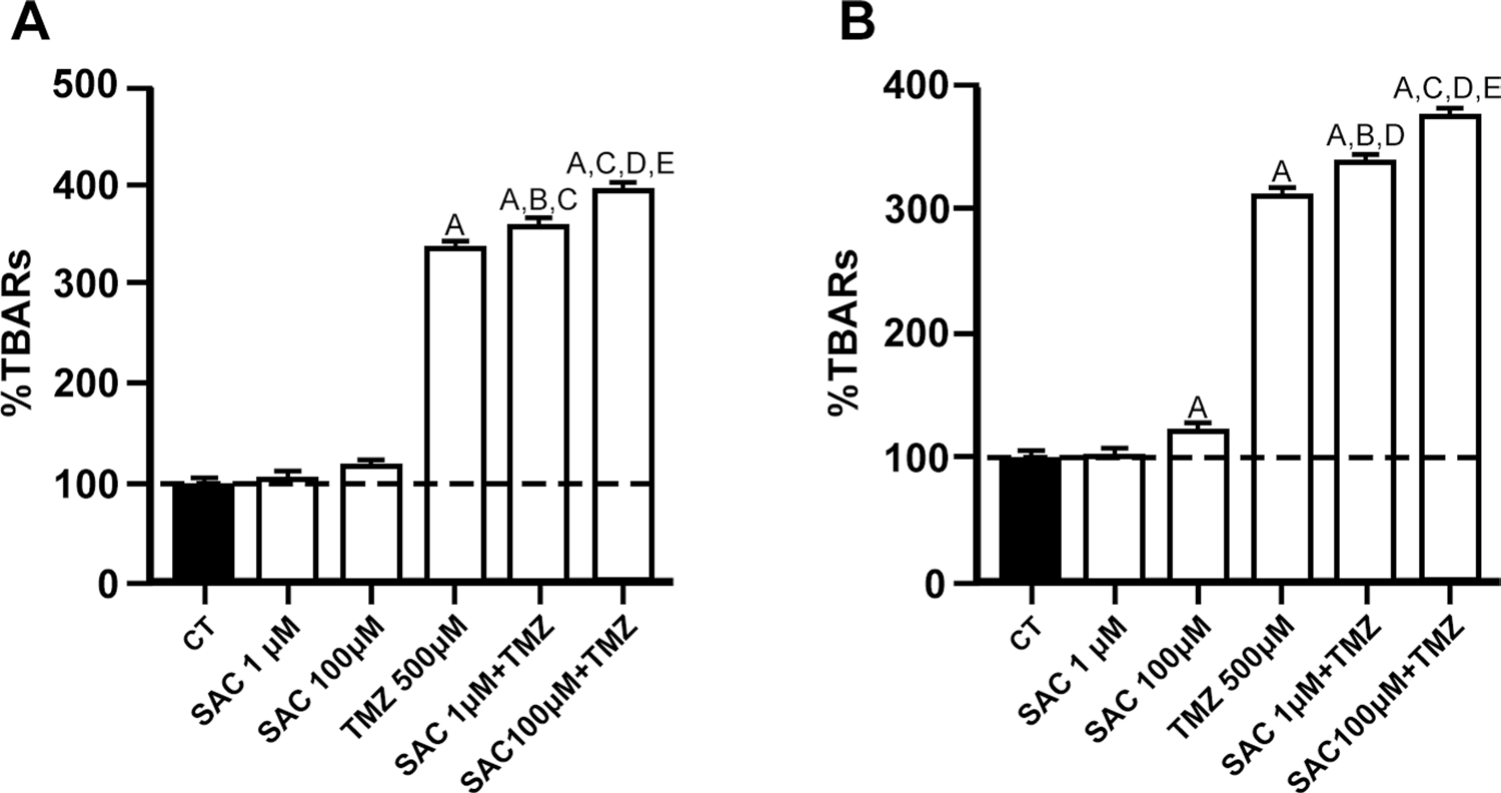
Effects of SAC, TMZ, and SAC + TMZ treatments on the levels of oxidative damage to lipids in RG2 and C6 cell lines. Cells were treated with SAC (1 µM and 100 µM) and/or TMZ (500 µM) for 48 h. Charts depict the percentage of lipid peroxidation (thiobarbituric acid-reactive substances (TBARS) formation) compared to the control of RG2 (**A**) and C6 (**B**) cells. Bars represent mean values ± S.E.M. of four experiments per group, each in duplicate. ^a^*p* ≤ 0.05 and ^A^*p* ≤ 0.01, different of control (PBS); ^B^*p* ≤ 0.01, different of SAC (1 µM); ^C^*p* ≤ 0.01, different of SAC (100 µM); ^D^*p* ≤ 0.01, different of TMZ; ^E^p ≤ 0.01 different of SAC + TMZ (1 µM + 500 µM). One-way ANOVA followed by Bonferroni’s test

Lipid peroxidation, detected by the TBA assay, was used in both RG2 and C6 cell lines to explore the effects of SAC, TMZ and SAC + TMZ on oxidative damage to lipids (Fig. 4). Results revealed that SAC (1 µM or 100 µM) did not increased lipid peroxidation in RG2 (Fig. 4A) compared to the control group. SAC (100 µM) induced a moderate, yet significant increase (*p* ≤ 0.01) in oxidative damage to lipids in C6 cells (Fig. 4B) cells, whereas TMZ (500 µM) readily augmented this marker in both cell lines by 217% (4A) and 235% (4B) above the control group (*p* ≤ 0.01), respectively. When the SAC and TMZ combinations (1 or 100 µM SAC + 500 µM TMZ) were tested, a significant SAC-concentration-dependent increase in lipid peroxidation was observed in both cell lines compared to TMZ alone (*p* ≤ 0.01).

### The combination of SAC + TMZ stimulates the decrease of GSH while augments GSSG in RG2 and C6 cell lines

**Fig. 5:**
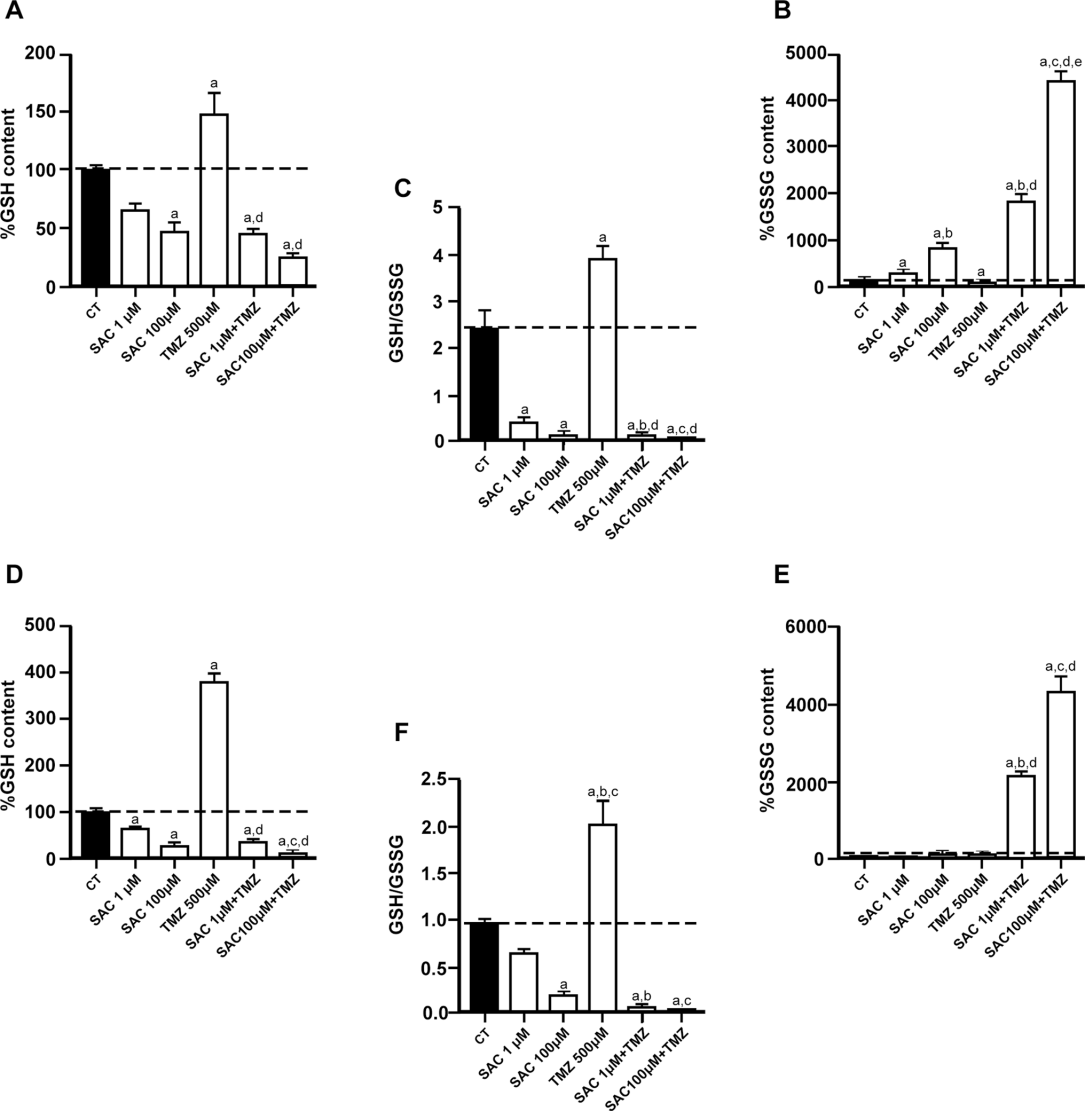
Effects of SAC, TMZ, and SAC + TMZ on the cellular levels of reduced glutathione (GSH, in **A** and **D**), oxidized glutathione (GSSG, in **B** and **E**), and the GSH/GSSG ratio (in **C** and **F**) in RG2 and C6 cell lines. Cells were treated with SAC (1 µM and 100 µM) and/or TMZ (500 µM) for 48 h. Charts depict the percentage of GSH, GSSG and GSH/GSSG ratio compared to the control of RG2 (**A-C**) and C6 (**D**–**F**) cells. Bars represent mean values ± S.E.M. of four experiments per group, each in duplicate. ^a^*p* ≤ 0.05, different of control (PBS); ^b^*p* ≤ 0.05, different of SAC (1 µM); ^c^*p* ≤ 0.05, different of SAC (100 µM); ^d^*p* ≤ 0.05, different of TMZ; ^e^*p* ≤ 0.05, different of SAC + TMZ (1 µM + 500 µM). One-way ANOVA followed by Bonferroni’s test

GSH and GSSG quantification was carried out in RG2 and C6 cell lines to confirm the changes in redox activity induced by the different treatments tested (Fig. 5). Results revealed that while 1 and 100 µM SAC significantly decreased GSH levels in RG2 (Fig. 5A) and C6 (Fig. 5D) cells compared to control values (*p* ≤ 0.05, in both cases), 500 µM TMZ significantly augmented this endpoint in both cell lines (*p* ≤ 0.05, compared to the control group). Interestingly, the combination of 1 or 100 µM SAC + 500 µM TMZ maintained GSH levels below the control, and even below SAC alone, both in RG2 (5A) and C6 (5B) cells, and these changes were statistically different from the control group (*p* ≤ 0.05) and the TMZ group (*p* ≤ 0.01).

Opposite changes were observed in the levels of GSSG in RG2 and C6 cells (Fig. 5B, and 5E, respectively), where the two concentrations of SAC moderately increased this marker in RG2, TMZ maintained basal levels of GSSG, and the combination of SAC + TMZ significantly increased this marker (*p* ≤ 0.01 in all cases, compared to the control) in a SAC concentration-dependent manner.

Subsequently, the GSH/GSSG ratio in RG2 (Fig. 5C) and C6 (Fig. 5F) cells showed a significant decrease for the two concentrations of SAC and the combination of SAC + TMZ (*p* ≤ 0.01), while this ratio was significantly increased in the TMZ treatment (*p* ≤ 0.01) compared to the control. Both cell lines displayed similar responses to the treatments, highlighting the fact that the tendencies of each compound were the same in RG2 and C6 cells.

### TMZ decreased the Nrf2/ARE binding activity in RG2 cells while SAC exhibited a biphasic effect on the TMZ-induced increase of Nrf2/ARE activity in C6 cells

**Fig. 6:**
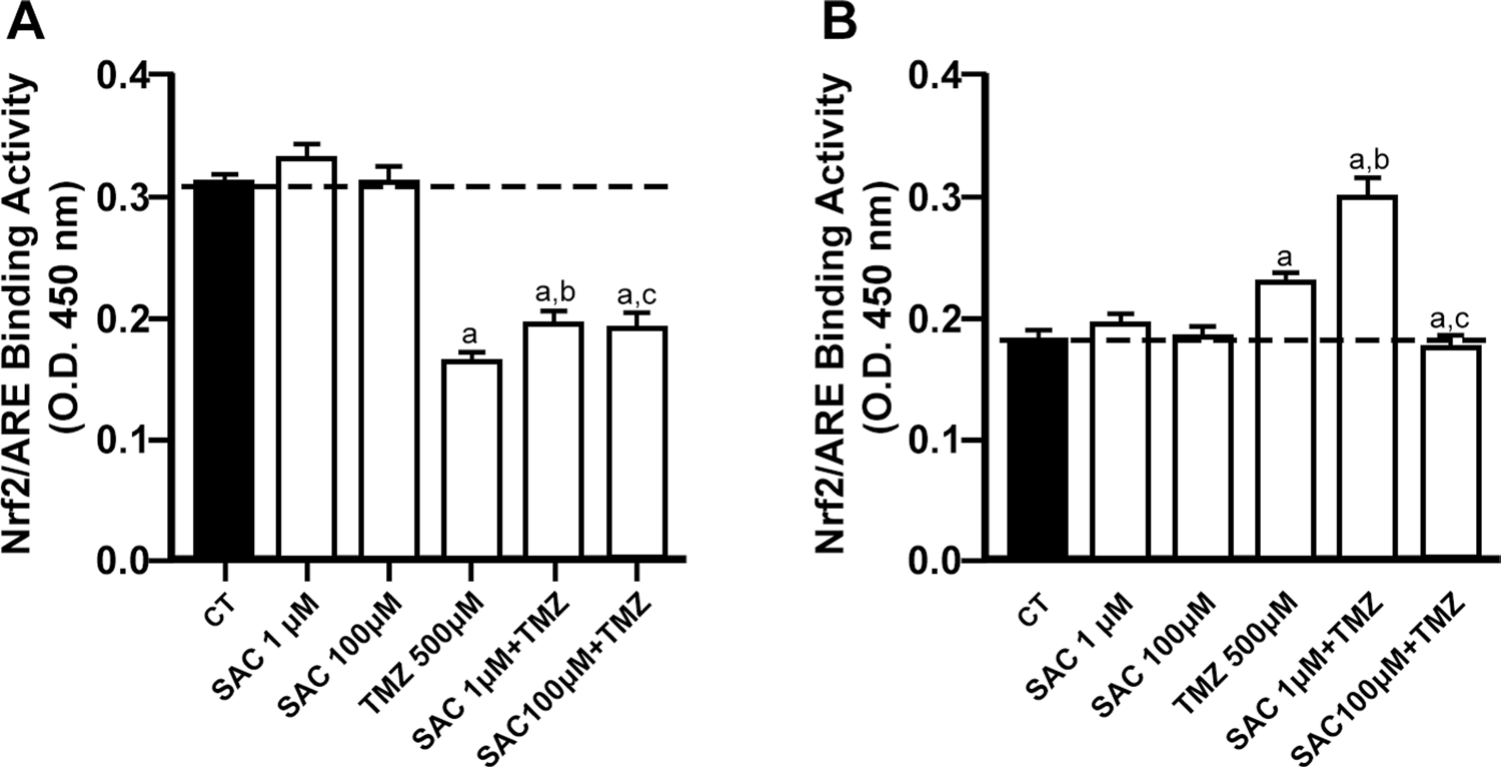
Effects of SAC, TMZ, and SAC + TMZ on the activity of the Nrf2/ARE complex in RG2 and C6 cell lines. Cells were treated with SAC (1 µM and 100 µM) and/or TMZ (500 µM) for 48 h. Charts depict the activity of Nrf2/ARE (optical density units) compared to the control in RG2 (**A**) and C6 (**B**) cells. Bars represent mean values ± S.E.M. of four experiments per group, each in duplicate. ^a^*p* ≤ 0.05, different of control (PBS); ^b^*p* ≤ 0.05, different of SAC (1 µM); ^c^*p* ≤ 0.05, different of SAC (100 µM); ^d^*p* ≤ 0.05, different of TMZ; ^e^*p* ≤ 0.05, different of SAC + TMZ (1 µM + 500 µM). One-way ANOVA followed by Bonferroni’s test

To determine whether the cytotoxic and antitumor effects of SAC and/or TMZ were linked to the regulation of Nrf2 activation, Nrf2/ARE binding activity was evaluated in both RG2 and C6 cells as a functional index of Nrf2 (Fig. 6). Neither 1 µM nor 100 µM SAC modified the basal Nrf2 activity in either cell lines. In contrast, in RG2 cells, TMZ decreased Nrf2 activity by ~ 50% compared to the control group (*p* ≤ 0.05; Fig. 6A), while increasing Nrf2 activity in C6 cells by ~ 27% compared to the control group (*p* ≤ 0.05; Fig. 6B), highlighting the different adaptive and compensatory responses of these cell lines to TMZ treatment. The co-administration of SAC + TMZ did not modify the effect of TMZ alone in RG2 cells (6A). In contrast, in C6 cells (6B), 1 µM SAC increased the effect of TMZ on Nrf2 activity by ~ 21% (*p* ≤ 0.05), while 100 µM SAC decreased the effect of TMZ by ~ 38% (*p* ≤ 0.05), thus revealing a biphasic effect.

## Discussion

Our results showed that both RG2 and C6 cell lines were sensitive to SAC and TMZ, displaying a concentration-dependent loss of cell viability and increased oxidative stress. Concerning TMZ, our results corroborate those by Liu et al. [35], who reported that rat RG2 cells showed significant sensitivity to resveratrol and TMZ in two human glioblastoma cell lines. For SAC, it has been reported that the effective concentrations for induction of cytotoxicity in bladder, neuroblastoma, breast, liver and lung cancer cells were in the range of µM to mM [36–42]. We showed that the cytotoxic effects of SAC can be achieved in tumor cells at micromolar concentrations, as SAC decreased cell viability in RG2 and C6 cells at lower range of concentrations. In general, both cell lines exhibited a similar response to SAC and/or TMZ, except for a slightly higher sensitivity of RG2 cells observed in some endpoints evaluated, and the response to Nrf2 activation when treated with SAC and/or TMZ. These differences might be due to intrinsic biological characteristics of each cell line, which may confer differential properties to tumor cells in response to pharmacological challenges in specific manners [43]. In fact, cell heterogeneity composing a given tumor growing in tissues is not entirely represented by individual cell lines [44]; therefore, different tumor cell lines develop differential responses to drugs in terms of magnitude and tendency, as their sensitivity is not tissue-specific [45].

TMZ exerts cytotoxic effects in human and rat glioblastoma cell lines. Concentrations of this compound significantly decreased cell viability at 100–750 µM [35, 46–49], corroborated herein, thus supporting the concept that TMZ affords an appropriate positive control of antineoplastic activity in our cell model. Interestingly, TMZ has been shown to transform into its active metabolite 5-(3-methyl-1-triazeno)imidazole-4-carboxamide (MTIC) through a hydrolysis reaction, and these structural modifications occur before the compound reaches the tumor [50]. In fact, TMZ efficacy can be affected by redox modifications in the tumor microenvironment.

In addition, experiments combining increased concentrations of SAC and TMZ were carried out to investigate whether these compounds exert additive cytotoxic effects. The combination of 100 μM SAC + 500 μM TMZ inhibited RG2 and C6 viability in an additive manner, suggesting the generation of reciprocal sensitization of cells to these compounds, which was dependent on SAC concentration. This result corroborates findings by Liu et al. [35], who demonstrated that the effects of TMZ on glioblastoma cells can be augmented by antioxidants co-administration.

Although protective effects of SAC have been linked to its antioxidant properties and the regulation of redox activity in normal cells and tissues [29, 51], we demonstrate herein that the cytotoxic effects of SAC were mediated by inverse redox regulatory properties as it altered the GSH/GSSG ratio in both cell lines. These findings agree with a previous report showing that SAC increases oxidative damage in lung cancer cells [52]. Therefore, it is likely that several antioxidants, including SAC and resveratrol, can induce these inverse effects (protective *vs.* cytotoxic) by modulating cellular redox systems.

Cancer cells are metabolically active and produce high levels of ROS, becoming particularly sensitive to the high levels of OS produced by compounds such as conventional chemotherapeutics [9]. TMZ and cisplatin are chemotherapeutic drugs used for GBM, and both have been reported to induce DNA damage and ROS formation, [53–56]. However, prolonged treatment with these drugs reduces the ROS content in cancer cells, inducing resistance to chemotherapeutic agents [57] through adaptive mechanisms regulated by the Nrf2/ARE axis [11]. This transcription factor is up-regulated or constitutively activated in several types of cancer [58–60]. The use of Nrf2 activity inhibitors derived from natural compounds can improve the efficacy of chemotherapeutic agents by detoxifying endogenous antioxidants, such as GSH, and increased drug excretion transporter expression [61, 62]. Our findings in RG2 cells corroborate the above, highlighting the ability of TMZ in sensitizing glioblastoma cells. This evidence agrees with previous reports showing that high concentrations of polyphenols (> 50 µM) can induce pro-oxidant effects, while suppressing antioxidant systems and inhibiting Nrf2 in tumor cells [63]. It has also been reported that inhibition of Nrf2 sensitizes cancer cells to the effects of chemotherapeutic drugs such as doxorubicin, oxaliplatin, and paclitaxel, inhibiting cell proliferation both in in vitro and in vivo conditions [64–67]. In turn, at 1 µM concentration, and in combination with TMZ, SAC increased Nrf2/ARE activity in C6 cells probably as an adaptive response, whereas at 100 µM, and in combination with TMZ, it returned this measurement to basal levels, exhibiting a biphasic effect. Notably, SAC per se did not change the basal activity of Nrf2/ARE in either RG2 or C6 cells. Regarding the more consistent effect of SAC + TMZ observed in RG2 over C6 cells, it is noteworthy that in contrast to C6 cells, RG2 cells failed to overexpress the Epidermal Growth Factor Receptor (EGFR), a protein known to be responsible for sustained downstream Nrf2 activation, which in turn may account for increased sensitivity to chemotherapeutic agents [68]. Therefore, despite SAC’s and TMZ’s induction of tumor cell damage in both cell lines, they likely use different signaling mechanisms (Nrf2 axis) to mediate these effects, requiring additional future studies.

GSH plays a major role in resistance to conventional chemotherapy, while promoting tumor recurrence. GSH participates in redox homeostasis [69], as it regulates antioxidant and thiol levels, modulating the activities of several redox-sensitive signaling molecules and transcription factors through S-glutathionylation [70]. GSH levels are increased in different tumor types [71], and this increase contributes to the resistance of tumor cells to chemotherapeutic drugs through the reduction of ROS levels, detoxification of several medications, and/or contributing to DNA repair [72]. In addition, metabolic enzymes related to GSH are overexpressed in GBM-resistant cells [73] and they regulate cell responses to chemotherapy [74, 75]. In contrast, GSH depletion or down-regulation of metabolic enzymes related to GSH reverses drug resistance, promoting the recovery of sensitivity to chemotherapeutic agents in resistant cells. Here, a significant decrease in GSH levels was induced by SAC alone and in combination with TMZ in both cell lines, consistent with Chen et al. [76]. Specifically, TMZ increased GSH levels in GBM cells, while its combination with erastin effectively reversed this effect. Moreover, an aged-garlic extract (AGE)-derived compound, allicin (40 µM), led to decreased basal GSH levels in leukemia cell lines [77] and colorectal cancer [78]. Furthermore, in an in vitro model of neuroblastoma exposed to SAC, intracellular GSH levels decreased [41], supporting the concept that combined administration of SAC and TMZ sensitizes glioblastoma cells through the modification of the redox status, leading to loss of cell viability. The antitumor effects of 50 µM SAC have been addressed in 2D- and 3D-cell culture models in C6 cells [79]. Changes in gene expression profiles that were different among the culture models, with decreased JAGGED1 and NOTCH gene expressions—typically altered in cancer cells—in the groups treated with SAC. Therefore, our study provides additional biochemical and molecular evidence to these findings.

The thiol ratio contributes to cell response to OS. For example, changes in NADPH/NADP^+^ and GSH/GSSG ratios are redox modulators that reduce pro-oxidant insults. High ratios are needed to preserve optimal cell redox potential [80, 81]. In addition, decreased GSH/GSSG ratio may induce apoptosis [82, 83] probably through the loss of Bcl-1 and activation of caspases. Conversely, its increase may have inverse effects [84], as evidenced by the findings here, where the levels of GSSG were significantly increased by combined treatment with SAC + TMZ in both cell lines, decreasing the GSH/GSSG ratio. Combined, this evidence suggests that in RG2 and C6 cells treated with SAC + TMZ, the cellular capacity to convert GSSG into GSH is deficient, probably due to decreased mitochondrial NADPH activity. This proposed mechanism agrees with the findings of a previous study [41] where both AGE and SAC exhibited cytotoxic effects in SJ-N-KP human neuroblastoma cells and MYCN-amplified IMR5 cells via altered redox status and induction of mitochondrial permeability transition.

Although under normal cellular conditions, antioxidants such as SAC play a detoxifying and protective role in cells, there is evidence that allylic compounds can induce pro-oxidant activity in tumor cells due to their structure and high reactivity. In fact, allylic compounds are the main source of disulfides, polysulfides, and protein thiols, thus mediating thiol/disulfide exchange through the induction of decreased levels of GSH and thiolation of reactive cysteine residues in proteins [85], resulting in OS in cells with deficient antioxidant defenses, such in cancer cells [86], ultimately leading to cell death [87]. Therefore, GSH, and the GSH/GSSG balance, play a crucial role in cell survival and cytotoxicity, and should be considered for the design of antitumor therapy and the reversion of drug resistance [72, 75].

**Fig. 7:**
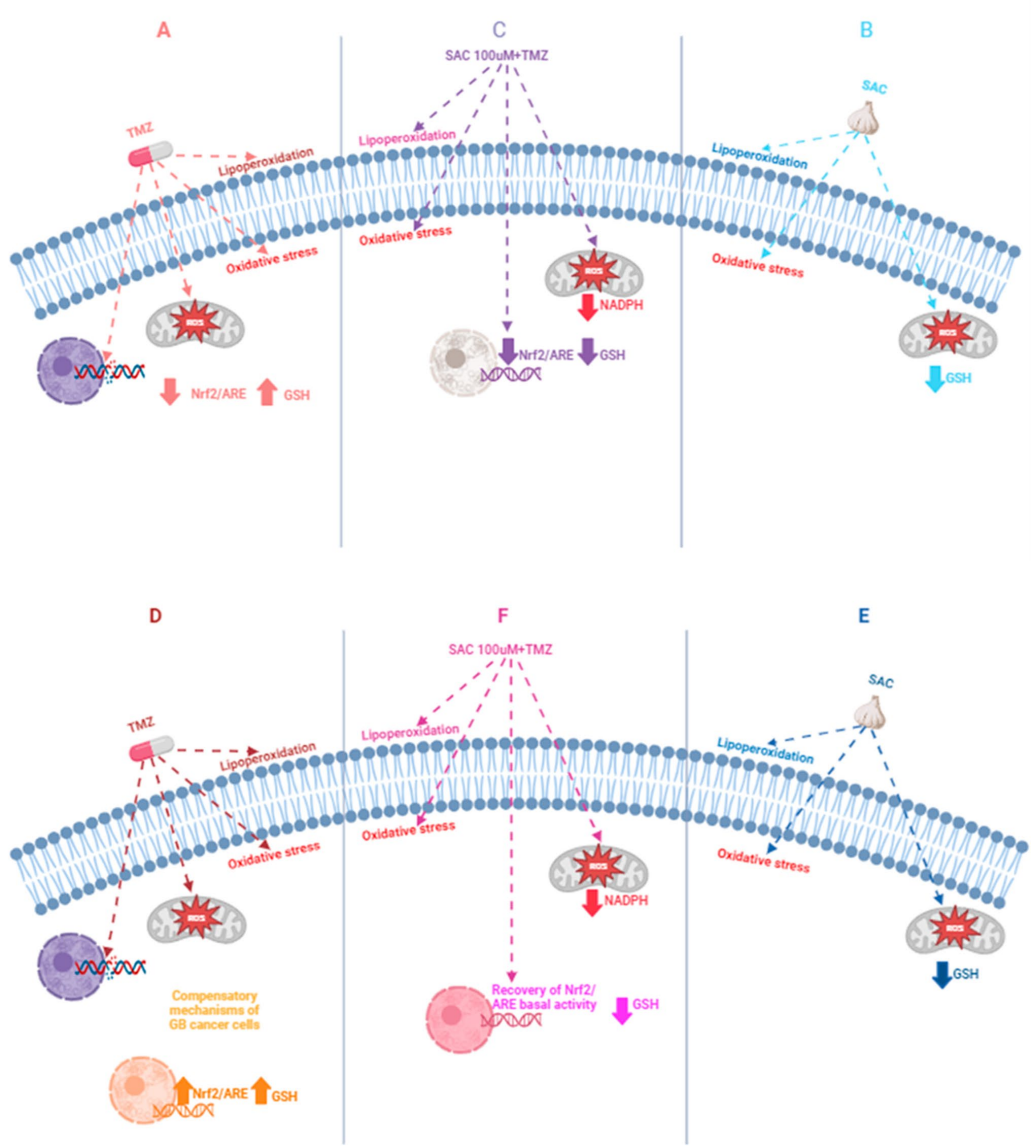
Schematic representation of the suggested cytotoxic mechanisms induced by TMZ, SAC + TMZ, and SAC in RG2 (upper panel) and C6 (bottom panel) glioblastoma (GBM) cell lines. In (**A**) and (**D**), TMZ induced cytotoxic effects through an increase in oxidative damage to lipids and a decrease in cell viability. However, to counteract these toxic events, both cell lines may display compensatory mechanisms, such as an increased GSH/GSSG ratio aimed to generate cell resistance to the compound. TMZ also decreases the binding activity of Nrf2/ARE in RG2 (**A**) to sensitize cells to oxidative damage, whereas as part of the compensatory response, C6 cells (**D**) may display adaptive responses such as Nrf2/ARE activity upregulation. In turn, SAC, at high concentrations, increases lipoperoxidation while decreasing the cell viability and the GSH/GSSG ratio in RG2 (**B**) and C6 (**E**) cells, thus leading to enhanced cytotoxicity in tumor cells. In (**C**) and (**F**), the combination of SAC + TMZ exerted an additive effect on cytotoxicity in both cell lines by increasing oxidative damage to lipids and decreasing cell viability and the GSH/GSSG ratio; however, differential regulatory signaling responses between tumor cell lines to SAC + TMZ point to a decrease in Nrf2/ARE binding activity in RG2 cells (**C**) as a sensitizing toxic mechanism in contrast to the preserved binding activity of Nrf2/ARE in C6 cells (**F**) as a possible compensatory response displayed by tumor cells

 The experimental evidence collected in this study and those described in literature demonstrate the cytotoxic and antitumor potential of SAC for the management of glioma cells. This suggests a promising scenario for its consideration in combined therapies with antitumor compounds such as TMZ. Finally, Fig. 7 summarizes the most relevant toxic events occurred in the toxic models investigated herein.

## Concluding remarks

We demonstrated that the antitumor and cytotoxic properties exerted by both SAC and TMZ on RG2 and C6 glioblastoma cells were linked to redox modifications, as evidenced by changes in the GSH/GSSG ratio, oxidative damage to lipids, and Nrf2 activity. Notably, the effects evoked by these compounds were additive when co-administered to cells, suggesting that SAC is a potential candidate for the design of combined therapy for the treatment of GBM, which is supported by the lack of toxic effect of this antioxidant on primary astrocytes (*Supplementary Material*). However, more detailed evidence at the molecular signaling level is needed to validate SAC as a novel candidate for GBM therapy.

## Supplementary Information

Supplementary material 1.

## Data Availability

The datasets used and/or analyzed during the current study are available from the corresponding authors upon reasonable request.
