# Supplementary material for: S-allyl-cysteine triggers cytotoxic events in rat glioblastoma RG2 and C6 cells and improves the effect of temozolomide through the regulation of oxidative responses
Source: Discov Oncol. 2024 Jul 8;15:272. doi: 10.1007/s12672-024-01145-3 (PMC11231126; doi:10.1007/s12672-024-01145-3)
Supplement: Supplementary file 1 — Supplementary material 1. [file 12672_2024_1145_MOESM1_ESM.pdf]

Supplementary material to "S-Allyl-Cysteine Triggers Cytotoxic Events in Rat Glioblastoma RG2 and C6 Cells and Improves the Effect of Temozolomide Through the Regulation of Oxidative Responses" for Discover Oncology

Carolina Y. Reyes-Soto[1,2], Ricardo J. Ramírez-Carreto[2,3,4], Luz Belinda Ortíz-Alegría[5], Alejandro Silva-Palacios[6], Cecilia Zazueta[6], Sonia Galván-Arzate[7], Çimen Karasu[8], Isaac Túnez[9], Alexey A. Tinkov[10,11,12], Michael Aschner[13], Tessy López-Goerne[14], Anahí-Chavarría[2,*], Abel Santamaría[14,15,*]

[1]Posgrado en Ciencias Biológicas, Universidad Nacional Autónoma de México, Mexico City 04510, Mexico.

[2]Unidad de Investigación en Medicina Experimental, Facultad de Medicina, Universidad Nacional Autónoma de México. Mexico City 06726, Mexico.

[3]Facultad de Química, Universidad Nacional Autónoma de México, 04510, Mexico, Mexico.

[4]Programa de Doctorado en Ciencias Biomédicas, Universidad Nacional Autónoma de México, Mexico City 04510, Mexico.

[5]Laboratorio de Inmunología Experimental, Subdirección de Medicina Experimental, Instituto Nacional de Pediatría, Secretaría de Salud, Mexico City 04530, Mexico.

[6]Departamento de Biomedicina Cardiovascular, Instituto Nacional de Cardiología *Ignacio Chávez*, SSA. Mexico City 14080, Mexico.

[7]Departamento de Neuroquímica, Instituto Nacional de Neurología y Neurocirugía *Manuel Velasco Suárez*, S.S, Mexico City 14269, Mexico.

[8]Department of Medical Pharmacology, Cellular Stress Response and Signal Transduction Research Laboratory, Faculty of Medicine, Gazi University, Ankara 06500, Turkey.

[9]Instituto de Investigaciones Biomédicas Maimónides de Córdoba (IMIBIC); Departamento de Bioquímica y Biología Molecular, Facultad de Medicina y Enfermería, Universidad de Córdoba; Red Española de Excelencia en Estimulación Cerebral (REDESTIM), Córdoba 14071, Spain.

[10]Laboratory of Molecular Dietetics, IM Sechenov First Moscow State Medical University (Sechenov University), Moscow 119435, Russia.

[11]Departament of Elementology, and Department of Human Ecology and Bioelementology, Peoples' Friendship University of Russia (RUDN University), Moscow 117198, Russia.

[12]Laboratory of Molecular Ecobiomonitoring and Quality Control, Yaroslavl State University, Yaroslavl 150003, Russia.

[13]Department of Molecular Pharmacology, Albert Einstein College of Medicine. Bronx, NY 10461, USA.

[14]Laboratorio de Nanotecnología y Nanomedicina, Departamento de Atención a la Salud, Universidad Autónoma Metropolitana-Xochimilco, Mexico City 04960, Mexico.

[14,15]Facultad de Ciencias, Universidad Nacional Autónoma de México. Mexico City 04510, Mexico.

*Corresponding authors: Abel Santamaría (absada@yahoo.com) and Anahí Chavarría (anahi.chavarria@facmed.unam.mx). Send correspondence in first place to Abel Santamaría. Facultad de Ciencias, Universidad Nacional Autónoma de México, Mexico City 04510, Mexico & Laboratorio de Nanotecnología y Nanomedicina, Departamento de Cuidado de la Salud, Universidad Autónoma Metropolitana-Xochimilco, Mexico City 04960, Mexico. E-mail address: absada@yahoo.com

This section contains information regarding the effects of SAC and TMZ on primary astrocyte cultures which were assessed for comparative purposes against glioblastoma cultures.

Methods

Primary Astrocytes Culture

Astrocytes were isolated from newborn (1 PND) Wistar rats. Briefly, offspring were euthanized by decapitation and cortical tissues were separated and collected. Following dissection, the collected cortical tissues were placed in sterile phosphate-buffered saline (PBS) and incubated with trypsin for 8 min. Samples were then diluted in high-glucose DMEM (Invitrogen Co., NY, U.S.A.) supplemented with FBS (Gibco, U.S.A.) and centrifuged at 1,200 rpm for 5 min. The collected pellets were resuspended with DMEM containing 0.4% DNAase, and a second centrifugation was performed at 1,200 rpm for 5 min. Pellets were then resuspended with DMEM supplemented with 10% FBS and 1% antibiotics. Samples were seeded in plates and incubated as described above for one week. The medium was replaced every 2 days, and once high confluency was reached, cells were exposed to the same treatments described in the main article (S-allyl cysteine or SAC, and/or temozolomide or TMZ). Cell viability was assessed in primary cultures using the MTT assay according to the description made to this method in the main article. The entire protocol was presented at the Universidad Nacional Autónoma de México and was accepted by the Admission Committee of the Biological Sciences Doctoral Program (Student's Registration Number 308289853).

Results

SAC Preserved/Increased the Cell Viability Decreased by TMZ in Primary Astrocyte Cultures

The effects of SAC, TMZ and SAC+TMZ were tested in rat primary astrocytes to investigate whether these compounds can induce toxic effects under the physiological conditions represented by these cells (**Figure 1**). SAC (1 µM and 100 µM) increased cell viability by 12% and 33% compared to the control group ($p \leq 0.05$ and $p \leq 0.01$, respectively), while, as expected, TMZ (500 µM) decreased this endpoint by 25% compared to the control ($p \leq 0.01$). In turn, co-administration of SAC (1 µM or 100 µM) and TMZ reversed the toxic effects induced by TMZ, improving cell viability by 15% and 44% above TMZ treatment, respectively ($p \leq 0.01$ in both cases). These results demonstrated that SAC is innocuous to astrocytes, while TMZ is toxic to these cells.

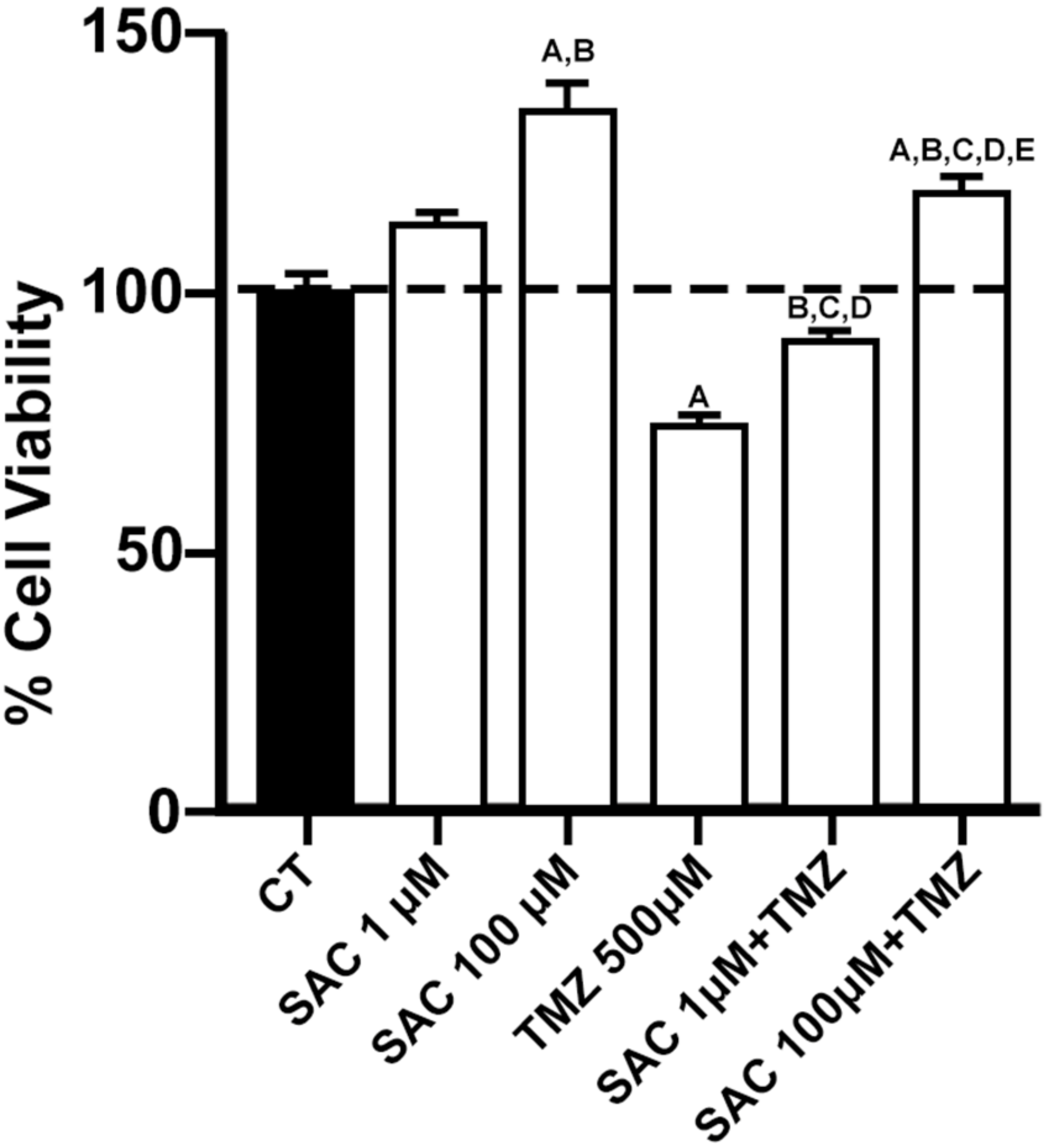

Supplementary Figure 1. Effects of SAC, TMZ and SAC+TMZ on cell viability in rat primary astrocyte cultures. Cells were treated with SAC (1 µM and 100 µM) and/or TMZ (500 µM) for 48 h. The chart depicts the percent of cell viability (compared to the control). Bars represent mean values ± S.E.M. of four experiments per group, each in duplicate. $^{a}p \leq 0.05$ and $^{A}p \leq 0.01$, different of control (PBS); $^{B}p \leq 0.01$, different of SAC (1 µM); $^{C}p \leq 0.01$, different of SAC (100 µM); $^{D}p \leq 0.01$, different of TMZ; $^{E}p \leq 0.01$, different of SAC (100 µM)+TMZ. One-way ANOVA followed by Bonferroni's test.

Discussion

A cell viability assay was carried out in primary astrocyte cultures to compare the effects of SAC and TMZ in RG2 and C6 cells vs. primary cells. While SAC *per se* did not affect cell viability of astrocytes at the concentrations tested, it protected cells against the TMZ-induced damage at both concentrations, thus demonstrating not only the toxic properties of TMZ (Liu et al. 2020), but also the innocuous nature of SAC under "normal" conditions and its protective capacity under toxic conditions (Orozco-Ibarra et al. 2016).

References

Liu Y, Song X, Wu M, Wu J, Liu J (2020) Synergistic effects of resveratrol and temozolomide against glioblastoma cells: underlying mechanism and therapeutic implications. Cancer Manag Res 12:8341-8354. https://doi.org:10.2147/CMAR.S258584

Orozco-Ibarra M, Muñoz-Sánchez J, Zavala-Medina Martín E, Pineda B, Magaña-Maldonado R, Vázquez-Contreras E, Maldonado P D, Pedraza-Chaverri J, Chánez-Cárdenas M E (2016). Aged garlic extract and S-allylcysteine prevent apoptotic cell death in a chemical hypoxia model. Biol Res 49:1-10. https://dx.doi.org/10.1186/s40659-016-0067-6
